# Blockade of Aquaporin 1 Inhibits Proliferation, Motility, and Metastatic Potential of Mesothelioma *In Vitro* but not in an *In Vivo* Model

**DOI:** 10.1155/2015/286719

**Published:** 2015-03-04

**Authors:** Sonja Klebe, Kim Griggs, Yuen Cheng, Jack Driml, Douglas W. Henderson, Glen Reid

**Affiliations:** ^1^Department of Anatomical Pathology, Flinders University, Adelaide, Bedford, SA 5042, Australia; ^2^SA Pathology, Department of Surgical Pathology, Flinders Medical Centre, Adelaide, Bedford, SA 5042, Australia; ^3^Asbestos Diseases Research Institute, Bernie Banton Centre, NSW 2139, Australia; ^4^School of Medicine, University of Sydney, NSW 2006, Australia

## Abstract

*Background*. Malignant mesothelioma (MM) is an aggressive tumor of the serosal membranes, mostly the pleura. It is related to asbestos exposure and has a poor prognosis. MM has a long latency period, and incidence is predicted to remain stable or increase until 2020. Currently, no biomarkers for a specific targeted therapy are available. Previously, we observed that expression of aquaporin 1 (AQP1) was an indicator of prognosis in two independent cohorts. Here we determine whether AQP1 inhibition has therapeutic potential in the treatment of MM. *Methods*. Functional studies were performed with H226 cells and primary MM cells harvested from pleural effusions. AQP1 expression and mesothelial phenotype was determined by immunohistochemistry. AQP1 function was inhibited by a pharmacological blocker (AqB050) or AQP1-specific siRNA. Cell proliferation, migration, and anchorage-independent cell growth were assessed. A nude mouse heterotopic xenograft model of MM was utilised for the *in vivo* studies. *Results*. Inhibition of AQP1 significantly decreases cell proliferation, metastatic potential, and motility without inducing nonspecific cytotoxicity or increasing apoptosis. *In vivo* blockade of AQP1 had no biologically significant effect on growth of established tumours. *Conclusions*. Targeted blockade of AQP1 restricts MM growth and migration *in vitro*. Further work is warranted to fully evaluate treatment potential *in vivo*.

## 1. Introduction

Malignant mesothelioma (MM) is an aggressive tumour of the serosal membranes. It most commonly affects the pleura and is related to past inhalation of asbestos. The minimum latency interval between the first exposure to asbestos and the discovery of the MM is in excess 10 years. Once considered a rare tumour [1], its incidence is projected to increase in most industrialised countries in 2015–2020 [2, 3]. Many recent cases are related to nonoccupational exposures (e.g., “handyman” exposures as a consequence of home renovation work) and, due to this type of exposure, increasingly younger patients are affected, some of whom sustained bystander exposures as children when their parents renovated. Based on conservative estimates of incidence, future economic liabilities are estimated to reach around $200 billion in the US, $80 billion in Europe, and AU$8 billion in Australia [4–6].

MM has an extremely poor prognosis, because available treatment strategies are limited. Radical surgery and combinations of radiotherapy, immunotherapy, and chemotherapy regimens are being trialed, but their benefits in terms of patient survival are unproven at this stage [7, 8]. In light of the limitations of current treatment strategies, new approaches and novel targets are needed urgently.

Aquaporins (AQPs), a family of at least 13 transmembrane water channel proteins, represent a potential target for cancer therapy [9]. They have roles in the normal cellular processes of water transport, proliferation, and pain perception [10–12] whereas, in cancer, AQP expression has been demonstrated to play a role in the growth and metastatic potential of a variety of tumours, including lung adenocarcinomas [9, 13–17].

AQP1 has also been demonstrated in the mesothelium of the pleura and peritoneum [18, 19], and a role of AQP1 for pleural water transport across mesothelial cells has been shown in a knockout mouse model [20]. We have previously demonstrated AQP1 by electron microscopy at the apical aspect of mesothelial cells, in keeping with that role in water transport [21].

AQP1 can be expressed by vascular endothelium as well as tumor cells, and blockade of AQP1 expressed by either cell types in the tumour may be beneficial. The direct effects of tumour-derived AQP1 expression on tumour growth have been demonstrated in other models: melanoma cells transfected with AQP1 showed increased growth and, in an* in vivo* model of melanoma, AQP1 blockade or inoculation of melanoma into AQP1-deficient mice slowed tumour growth by limiting vascular supply [22, 23]. It has been suggested that modulation of tumour cells, either by specific AQP inhibitors/blockers and agonists or by indirect modulation of closely associated growth factors, may become a future treatment strategy for some tumours [24, 25]. However, there was insufficient knowledge about the functions of AQP1 in MM.

We have reported previously that AQP1 expression by MM tumour cells is an independent prognostic factor for improved survival times in malignant mesothelioma: higher levels of AQP1 expression by* tumour cells only* (i.e., not vascular cells) predict improved survival [21]. We postulated that higher AQP1 expression in these tumours may reflect better differentiation, since normal mesothelium expresses AQP1. We hypothesised that the retained expression of AQP1 in this tumour may offer treatment potential to AQP1-expressing tumours. MM characteristically grows by direct spread along the pleural surface, where it forms nodules on the pleural surface. This is thought to relate to the sliding motion of tumour cells. AQP1 facilitates movement of both endothelial cells and some tumour cells [23, 26–29]. Inhibition of this type of cell motility/tumour growth especially may improve patient survival.

We therefore investigated the role of AQP1 on MM cell growth and movement and the effects of AQP1 blockade on MM cells* in vitro* and* in vivo* using a heterotopic nude mouse model.

## 2. Methods

### 2.1. Harvesting of Primary MM Cells from Pleural Effusions

Cells from 15 separate pleural effusion fluids from 13 patients (12 male and one female), aged between 65–94 years diagnosed with MM, were harvested. Two male subjects each contributed two effusions to the study. The tumours were diagnosed as epithelioid (12) and biphasic (3) subtypes. The work was approved by the Southern Adelaide Clinical Human Research Ethics Committee (approval number 381.09).

### 2.2. Cell Culture

Pleural effusion specimens were centrifuged at 500 ×g for 10 min at 25°C. The entire cell pellet was placed into culture with complete DMEM (10% fetal calf serum, 50 U/mL penicillin, and 50 *μ*g/mL streptomycin; Life Technologies, NY) and incubated at 37°C with 5% CO_2_. Culture medium was changed regularly, and the primary cells were assessed by a panel of immunohistochemical markers (Cam5.2, CK5/6, calretinin, D2-40 HBME-1, WT1, and negative markers BG8, TTF1, CD15, and BerEP4) to confirm their mesothelial phenotype. The commercially available H226 cell line (ATCC, Manassas, VA) was directly obtained from the ATCC and cultured in complete DMEM for less than six months. In addition, we confirmed mesothelial phenotype by 2-month immunohistochemical analysis and electron microscopy at the start of the experiments demonstrating complex branching microvilli. Pleural effusion MM cells were only used in subsequent experiments if they were determined to have >80% purity as assessed by immunohistochemistry (see below).

### 2.3. Immunohistochemical Analysis

Cells harvested from pleural effusions were resuspended in 1–3 drops of sheep plasma. A drop of thrombin (0.5 mL thrombin plus 9.5 mL 0.1 M CaCl_2_) was added to make a solid pellet, which was fixed with 10% formalin, processed with graded concentrations of ethanol and isopropanol, and then embedded in paraffin. Sections were cut 4 *μ*m thick, deparaffinised, and rehydrated prior to quenching with 1% H_2_O_2_. AQP1 immunohistochemistry was performed as previously described [21]. Calretinin and CAM5.2 immunohistochemistry was performed with citric acid retrieval prior to the addition of primary antibody (Calretinin; 1/5000; Invitrogen, CAM5.2, 1/1000; BD Australia). Both methods utilized the Novo Link polymer (Leica Microsystems, Wetzlar, Germany) and DAB + Chromogen (Dako, CA, USA) detection systems prior to hematoxylin counterstaining.

### 2.4. AQP1 Blocker

The AQP1 blocker (AqB050, US patent 7,906555B2) was purchased from Prof. Andrea Yool (University of Adelaide, Adelaide, SA 5000, South Australia). Toxicity was assessed using the ApoTox-Glo Triplex Assay (Promega Madison WI) according to the manufacturer's instruction to assess viability, cytotoxicity, and apoptosis events, with no toxicity identified at the tested concentrations.

### 2.5. siRNA Treatment

The H226 or primary MM cells were plated at 2.5 × 10^3^ cells/well and 5 × 10^3^ cells/well, respectively, in a 96-well plate before being reverse-transfected with 25 nM siRNA, as per manufacturer's instructions (Silencer Select siRNA, Applied Biosystems, Life Technologies). Briefly, siRNA (AQP1, GAPDH and siRNA Negative Control) was complexed with 0.125 *μ*L Lipofectamine RNAimax in 50 *μ*L of Opti MEM 1 reduced serum media for 10 min before cell dilutions were added (Life Technologies, NY). Medium was changed after 24 h and cells cultured until vector controls were confluent (4 days for H226 and 5 days for primary cells) prior to being used in proliferation experiments. The knockdown of genes by siRNA was confirmed with RT-PCR and immunohistochemistry.

### 2.6. Quantitative RT-PCR

AQP1 and GAPDH knockdown was confirmed by qRT-PCR. RNA extraction was performed using the RNeasy Mini Kit (QIAGEN); contaminating DNA was removed using Turbo DNA-free (Ambion, Life Technologies) before cDNA synthesis using the Superscript III First-Strand Synthesis system (Invitrogen, Life technologies, NY) as per manufacturer's instructions. AQP1 and GAPDH qRT-PCR were performed using TaqMan Gene Expression Assay system according to manufacturer's protocol (Life Technologies, NY). Peptidylprolyl isomerase A (PPIA) was used as a housekeeping gene. AQP1 mRNA was routinely knocked down to 6–17% of the vehicle control levels.

### 2.7. Cell Proliferation

Cell proliferation was assessed using the CellTiter 96 AQ_ueous_ Nonradioactive Cell Proliferation Assay (MTS) kit (Promega Corporation, Madison, WI). Briefly, H226 cells or primary cells were seeded in triplicate at 1.6 × 10^4^ cells/well in a 96-well plate and were incubated for 24 h prior to cell proliferation test. For AQP1 blocker experiments, cells were cultured for 23 h; then they were incubated with control medium (DMEM alone or DMEM plus DMSO) or medium containing the AQP1 inhibitor AqB050 for 1 h prior to the cell proliferation test. The CellTiter 96 Aqueous One Solution Reagent was added to each well and the plate was incubated at 37°C in a humidified, 5% CO_2_ incubator for 2 h before the absorbance (490 nm) was measured.

### 2.8. Anchorage-Independent Assay

H226 cells (2 × 10^4^ cells/mL) were suspended in DMEM containing 20% FBS and 3% agarose (Life Technologies, NY) with 20, 40, or 80 *μ*M AQP1 blocker or an equivalent volume of DMSO (vehicle control). The cell suspension (2 mL) was seeded into a 6-well plate on top of a solid layer of 4 mL DMEM containing 6% agarose and 10% FBS. Each treatment was in duplicate and data is from 3 separate experiments. Dishes were incubated for 3 weeks at 37°C with 5% CO_2_, until colonies were visible to the eye. Cells were stained with 0.2% crystal violet and were counted using a brightfield microscope. To determine colony size, 10 images were captured per well using AnalySIS getIT software and F-view camera attached to an IX71 inverted fluorescent scope with a 20x objective. Photos were analysed using ImageJ software.

### 2.9. Scratch Assay

H226 cells were seeded overnight at 1.5 × 10^5^ cells/well in a 24-well ImageLock microplate (Essen Bioscience, Michigan, USA). A scratch was made through the cell monolayer using a 4-pin WoundMaker (Essen Bioscience). After a wash with RPMI medium, DMSO or the AQP1 blocker was added in RPMI medium as indicated. Cell migration was monitored by the Incucyte Kinetic Imaging System (Essen Bioscience) every 2 h, up to 24 h after the scratch. Representative cell scatter images were created by tracking 10 evenly spaced cells (5 from each side of the wound) on an *x*/*y* axis from the images taken over the first 12 h. ImageJ was used to measure *x*/*y* location between the images and the points were plotted using Excel.

### 2.10. Animal Model

A subcutaneous xenograft model of MM was used as described previously [30, 31]. H226 cells were harvested, washed twice with PBS, and resuspended at 1 × 10^7^ cells/mL in PBS. From this cell suspension, 100 *μ*L was injected subcutaneously with a 25 G needle on a single occasion in the hind flank of BALB/c nude mice. In the initial dose-finding experiment, three groups of animals were examined: (1) mice treated with DMSO by daily intratumoural injection (*n* = 5), (2) mice treated with AQP1 blocker at 20 *μ*M by daily intratumoural injection (*n* = 6), and (3) mice treated with AQP1 blocker at 80 *μ*M by daily intratumoural injection (*n* = 6). Injections were performed daily for 5 days, once tumours reached ~50 mm^3^. In the follow-up experiment, three groups of animals were examined: (1) untreated control mice (*n* = 6), (2) mice treated with AQP1 blocker at 20 *μ*M by daily intratumoural injection (*n* = 5), and (3) mice treated with DMSO by daily intratumoural injection (*n* = 7). The AQP1 blocker was injected daily once the tumours had reached ~100 mm^3^. For this group, animals were kept until tumour size reached 500 mm^3^. All animals were euthanised by CO_2_ inhalation. Tumour width and length were measured with calipers and used to calculate tumour volume with the following formula: *V* = length × width^2^ × 0.52. Tumour growth was measured every 3 days. At the conclusion of the experiments, the resulting xenografts were excised, weighed, and processed for histological assessment. The work was approved by Flinders University and Southern Adelaide Local Health Network Animal Welfare Committee (approval number 805/12).

### 2.11. Statistical Analysis

For the* in vitro* work, Pearson's correlation was used to analyse cell proliferation* versus* AQP1 expression. Curve estimation determined that the data fit a quadratic model. Changes in proliferation and anchorage-independent growth of MM following addition of AqB050 or siRNA were evaluated by ANOVA with Tukey's post hoc analysis or by an independent samples *t*-test. If data could not be normalised it was analysed with the Friedman test with Wilcoxon signed-rank post hoc analysis. The wound healing assay and animal studies were analysed using a repeated measures ANOVA with Tukey's post hoc analysis. All analyses were performed using SPSS for Windows version 17.0.

## 3. Results

### 3.1. Increased AQP1 Expression Is Associated with Cellular Proliferation

**Figure 1:**
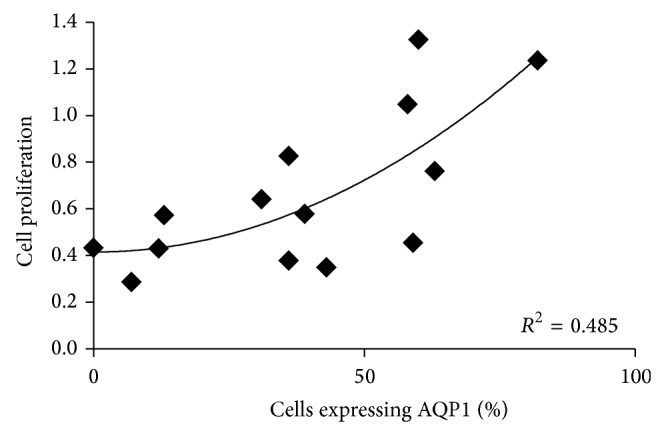
Higher AQP1 expression in primary MM cells correlates with increased proliferation rates. Primary MM cells were seeded at 1.6 × 10^4^ cells/well and were allowed to grow for 24 h. Cell proliferation was measured by MTS assay, *n* = 14. Curve fit determined a quadratic relationship. Analysed by Pearson's correlation, *R* ^2^ = 0.485; *P* = 0.026.

Primary MM cells harvested from pleural effusion fluids were cultured and proliferative activity was assessed by MTS assay. Higher levels of AQP1 expression were associated with higher rates of cell proliferation (*R* ^2^ = 0.485; *P* = 0.026; Figure 1).

### 3.2. Alteration of AQP1 Function and MM Cell Proliferation* In Vitro*

**Figure 2:**
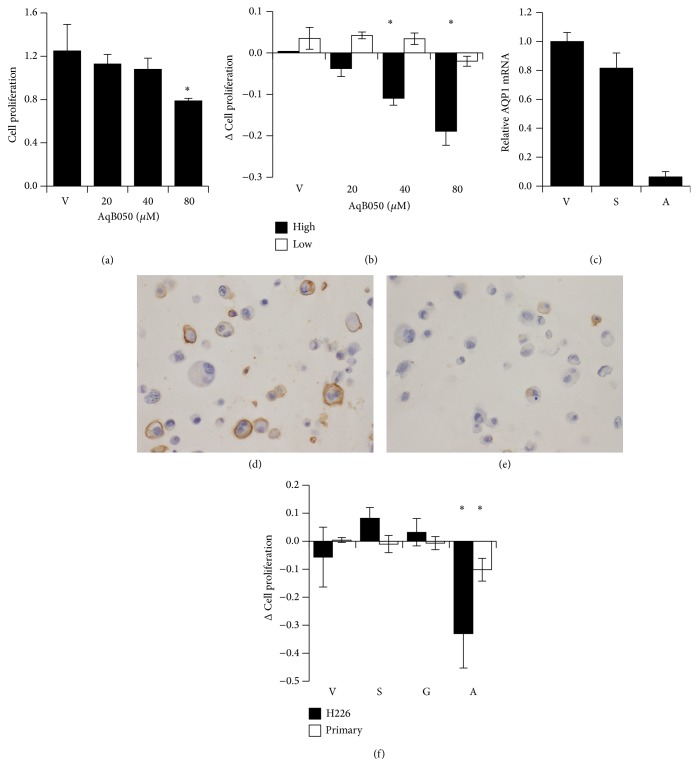
Decreased AQP1 function reduces the rate of cell proliferation. (a) H226 cells treated with AqB050 show dose-dependent decrease in proliferation, *n* = 3. Error bars: SD. Analysed by ANOVA, *P* = 0.021, with Tukey's post hoc analysis, *P* = 0.016 (as indicated by ∗). (b) Primary cells treated with AqB050 show dose-dependent decrease in proliferation only in those populations where ≥20% of the MM population expressed AQP1 (designated “high”); high *n* = 8 and low *n* = 3. Error bars: SEM, analysis by one-way ANOVA (high *P* < 0.001; low *P* = 0.397). (c) AQP1 siRNA treatment decreased AQP1 mRNA levels in primary MM cells and H226 cells. Decrease of AQP1 mRNA levels following siRNA knockdown was confirmed by RT-PCR. AQP1 mRNA was routinely decreased to 6–17% of the vehicle control levels. Shown is a representative example of primary MM cells. (d) AQP1 protein expression as assessed by immunohistochemistry (brown labeling) is preserved on the cell surface of primary MM cells treated with scrambled siRNA. (e) AQP1 protein expression as assessed by immunohistochemistry (brown labeling) is decreased on the cell surface of the same primary MM cells treated with AQP1 siRNA. (f) AQP1 siRNA treatment decreases proliferation of H226 and primary MM (H226: *n* = 3, one-way ANOVA *P* = 0.002, vector versus AQP1 siRNA *P* = 0.021; primary cells: *n* = 3, one-way ANOVA *P* = 0.006, vector versus AQP1 siRNA *P* = 0.008) with significance indicated by ∗. V: vector, S: scrambled, G: GAPDH, A: AQP1.

To determine whether AQP1 plays a functional role in MM cell proliferation, H226 cells and primary MM cells were cultured in control media or in the presence of the AQP1 blocker AqB050. The H226 cell line shows expression of AQP1 in >20% of tumour cells. There was a dose-dependent decrease in proliferative activity when cells were incubated with AqB050 for 1 h and, at 80 *μ*M, there was a statistically significant decrease in proliferative activity compared to the vector control (*P* = 0.021; Figure 2(a)).

Primary MM cell cultures where <20% of the cells expressed AQP1 by IHC were designated as AQP1-low, whereas in AQP1-high cultures ≥20% of the population exhibited AQP1 expression. There was a statistically significant dose-dependent decrease in cell proliferation when AQP1-high cells were treated with increasing doses of AqB050 (*P* < 0.001; Figure 2(b)). However, the AQP1-low cells showed no statistically significant difference in proliferation when exposed to AqB050 (*P* = 0.397; Figure 2(b)), regardless of the concentration of the blocker used.

To exclude a nonspecific effect of AqB050 and to confirm that the decrease in proliferation was in fact due to its action on AQP1, we transfected H226 cells and primary MM cells with AQP1 siRNA prior to measuring cell proliferation. Like the pharmacological AQP1 blocker, the AQP1 siRNA treatment significantly decreased cell proliferation rate in both the H226 cells (*P* = 0.021) and the primary cells (*P* = 0.008, Figure 2(f)). The scrambled siRNA (H226, *P* = 0.283; primary, *P* = 0.928) and GAPDH siRNA (H226, *P* = 0.612; primary, *P* = 0.962) treatments did not result in decreased cell proliferation. The specificity of the AQP1 siRNA treatment was demonstrated by decreased expression of AQP1 mRNA (Figure 2(c)) and protein (Figures 2(d) and 2(e)).

### 3.3. Blockade of AQP1 Decreases Cell Motility and Anchorage-Independent Growth

**Figure 3:**
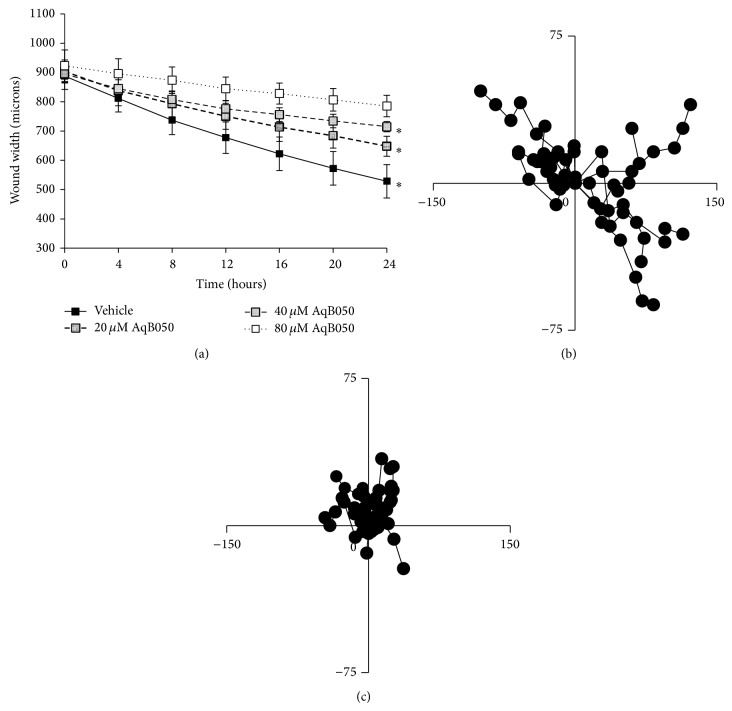
AQP1 blockade decreases cell motility of H226 cells in a wound-healing assay. (a) Migration of cells in a scratch assay is decreased in H226 cells treated with AqB050. Vehicle, *n* = 4; 20 *μ*M AqB050, *n* = 4; 40 *μ*M AqV050, *n* = 3; 80 *μ*M AqB050, *n* = 3. Analysis by repeated measures ANOVA *P* = 0.001 with Tukey's post hoc analysis (20 *μ*M *P* = 0.03, 40 *μ*M *P* = 0.01, and 80 *μ*M *P* = 0.001; significance indicated by ∗); error bars: SD. The position of 10 individual cells was tracked over 12 h by time-lapse photography. (b) Vehicle control medium. (c) 80 *μ*M AqB050 treatment.

**Figure 4:**
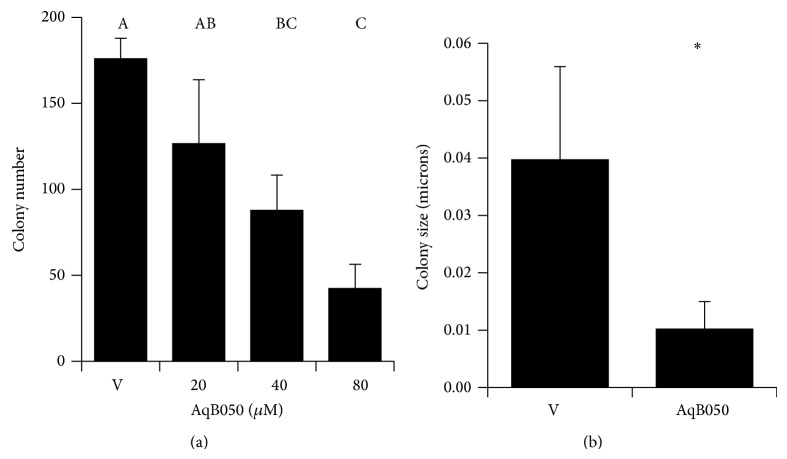
AQP1 blockade in H226 cells results in reduced colony number and size in an anchorage independent assay. (a) Number of colonies: *n* = 3, *P* = 0.001. Analysed by one-way ANOVA followed by Tukey's post hoc analysis (A, B, and C indicate significant subgroups, *P* ≤ 0.05). (b) Size of cell colonies: *n* = 3, *P* = 0.039. Analysed by independent samples *t*-test. Significant differences indicated by ∗. Error bars: SD. V: DMSO vehicle control.

A scratch assay was used to determine whether AQP1 blockade decreases MM cell motility. The ability of MM cells to move to cover the scratch wound was decreased in all AqB050-exposed MM groups in a dose-dependent manner (Figure 3(a), *P* ≤ 0.001). The position of single cells was tracked by time-lapse photography over 12 h, and cells treated with 80 *μ*M AqB050 showed less individual cell movement compared to cells in vehicle control (Figures 3(b) and 3(c)). An anchorage-independent growth assay, an indicator of metastatic growth potential, was performed. When H226 cells were incubated with increasing concentrations of AqB050, colony formation was inhibited in all treatment groups compared to the vehicle control (*P* = 0.001; Figure 4(a)). In addition to the smaller number of colonies, the average colony size in the 80 *μ*M treatment group was significantly smaller when compared to the vehicle control (*P* = 0.039; Figure 4(b)).

### 3.4. Subcutaneous Xenograft Model of MM

**Figure 5:**
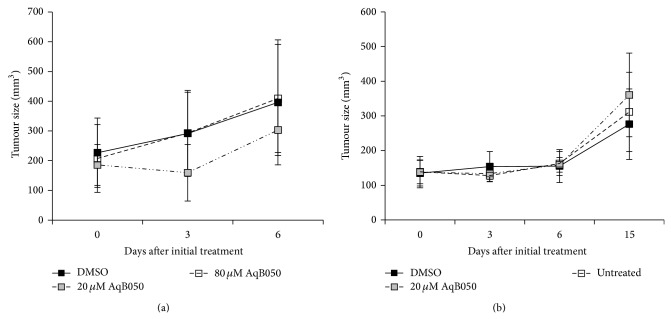
AQP1 blockade results in no difference in tumour size in H226 tumours in a subcutaneous xenograft model of MM. (a) A pattern towards an early decrease in tumour size was noted in the initial dose-finding experiment: DMSO, *n* = 5; 20 *μ*M AqB050, *n* = 6; 80 *μ*M AqB050, *n* = 6, *P* = 0.334. (b) A longer follow-up experiment using the dose with the highest potential for improved response confirmed no significant difference in tumor growth: untreated, *n* = 6; 20 *μ*M AqB050, *n* = 5; DMSO, *n* = 7, *P* = 0.761. Analysis by repeated measures ANOVA; error bars = SEM.

A pilot study using a BALBc/nude mouse subcutaneous xenograft model of MM was used to assess the therapeutic potential of AqB050* in vivo* and to determine a dose for future studies. Two doses (20 *μ*M and 80 *μ*M) were tested. There was no significant effect of either treatments on tumour size after six days (*P* = 0.334). However, tumours treated with 20 *μ*M AqB050 appeared to have a different growth pattern, with tumours appearing smaller than the vehicle-treated tumours at 3 days (Figure 5(a)). To further assess this impression, a second pilot study was conducted in the same animal model to see if this effect was reproducible or if there might be a more significant growth difference over a longer treatment period. However, after 15 days of treatment, there was no significant effect of treatment on tumour growth between treated, untreated, and vehicle controls (*P* = 0.761; Figure 5(b)).

## 4. Discussion

AQP1 is expressed by normal mesothelium. Expression of AQP1 is an independent prognostic factor in MM, with high levels of AQP1 expression correlating with increased survival in MM patients [21, 22]. Here we demonstrate for the first time that increased AQP1 expression correlates with increased cell proliferation in primary MM cells. This is in line with published observations in malignant melanoma, where increased AQP1 expression also correlated with faster growth [23, 26]. We postulate that retained AQP1 expression in MM indicates a more differentiated phenotype of the tumour, with some retained expression of surface proteins, which could be exploited for therapy. Therefore, because expression of AQP1 can be easily assessed in biopsy samples, this represents an attractive potential treatment for at least a subset of MM patients, as therapy could be specifically tailored depending on AQP1 expression levels. However, whilst* in vitro* work was promising,* in vivo* pharmacological blockade of AQP1 did not have a significant effect.

Exposure of MM of both cell lines and primary MM cells with a pharmacological AQP1 blocker, AqB050, resulted in significant decrease in MM cell proliferation at a concentration well outside the toxic range. The specificity of the effect being due to AQP1 blockade was confirmed by transfecting both H226 cells and primary cells with AQP1 siRNA, which resulted in comparable decrease of cell proliferation compared to the controls. Further evidence supporting the specificity of the effect being due to AQP1 blockade was provided by the proliferation rates of those primary MM cells expressing low levels of AQP1 not being affected by the AQP1 blocker. These results are similar to those found in lung cancer cells, where AQP1 overexpression was also associated with increased proliferation [15].

We also demonstrated that AQP1 blockade* in vitro* decreased cell movement in MM, as assessed in a wound-healing assay with increasing AqB050 concentrations as well as equivalent vehicle control treatments. In addition, we have tracked motility of individual cells over a 12-hour period, showing that individual cell motility was decreased. These findings support previous observations in other tumour models, where the ability of melanoma and breast cancer cells to migrate in a transwell assay and cover a wound in a scratch assay was significantly improved by transfection with an AQP1 expression construct [23]. Similarly, targeted disruption of AQP1 expression decreased cell motility in a malignant melanoma model [26]. We have shown here for the first time that, as in melanoma and breast cancer, AQP1 also promotes cell migration in MM. The reduction of colony number and size of MM when cultured in the presence of an AQP1 blocker in an anchorage-independent assay further supports our findings that AQP1 expression in MM contributes to cell motility. In melanoma cells this contribution to motility has been linked to expression of *β*-catenin, and a proportion of MM do express *β*-catenin [29, 32].

The ability of melanoma cells to form colonies in an anchorage-independent assay correlated with the ability of the same cells to produce metastases in an* in vivo* murine model [26]. For MM, pharmacological blockade of AQP reduced growth parameters* in vitro* in a similar fashion as was observed in melanoma with siRNA treatment but, unlike the melanoma model,* in vivo* therapy did not result in a significant alteration of tumour growth [26]. This could be due to a number of factors. It may be that the subcutaneous xenograft model does not model some of the aspects of the growth of mesothelioma well, such as the “sliding” motion of tumour cells, which is an important part of tumour growth in the native pleural space environment but which may not be modeled well subcutaneously and which is not a significant factor in melanoma. Alternatively, it is possible that the drug may have interacted with blood proteins, limiting bioavailability. Other factors include the type of drug administration (local instead of systemic), dose (a relatively small dose was used), and frequency of therapy (animals were injected daily). Also, the* in vivo* half-life of AqB050 has not been characterised, and it is possible that the local drug concentration did not reach therapeutic levels for long enough. Finally, AQP1 is also expressed on some blood components and vascular endothelium, so some of the drug could have been bound here, preventing it from reaching therapeutic concentrations at the tumour cell level.

We have demonstrated here for the first time that AQP1 has a functional role in MM proliferation, movement, and anchorage-independent growth. We have also demonstrated that it is possible to decrease growth and movement of MM* in vitro* by specific blockade of AQP1 using a pharmacological blocker, verifying that the effect was due to interference with AQP1 by siRNA. Our* in vitro* results suggest that AQP1 blockade may be a useful strategy to limit MM tumour growth, but further* in vivo* work is required to fully evaluate the potential of AQP1 blockade to contribute to therapy in MM.
